# Impairing proliferation of glioblastoma multiforme with CD44+ selective conjugated polymer nanoparticles

**DOI:** 10.1038/s41598-022-15244-0

**Published:** 2022-07-15

**Authors:** Dorota Lubanska, Sami Alrashed, Gage T. Mason, Fatima Nadeem, Angela Awada, Mitchell DiPasquale, Alexandra Sorge, Aleena Malik, Monika Kojic, Mohamed A. R. Soliman, Ana C. deCarvalho, Abdalla Shamisa, Swati Kulkarni, Drew Marquardt, Lisa A. Porter, Simon Rondeau-Gagné

**Affiliations:** 1 Department of Biomedical Sciences, University of Windsor, 401 Sunset Ave., Windsor, ON N9B 3P4 Canada; 2 Department of Chemistry and Biochemistry, University of Windsor, 401 Sunset Ave., Windsor, ON N9B 3P4 Canada; 3 Department of Neurosurgery, Faculty of Medicine, Cairo University, Cairo, Egypt; 4 Department of Neurosurgery, Jacobs School of Medicine and Biomedical Sciences, University at Buffalo, Buffalo, NY USA; 5 Schulich School of Medicine and Dentistry, Western University, London, ON Canada; 6 Department of Neurosurgery, Henry Ford Hospital, Detroit, MI 48202 USA; 7 Department of Physics, University of Windsor, 401 Sunset Ave., Windsor, ON N9B 3P4 Canada

**Keywords:** Biotechnology, Cancer, Neuroscience, Chemistry, Materials science, Nanoscience and technology

## Abstract

Glioblastoma is one of the most aggressive types of cancer with success of therapy being hampered by the existence of treatment resistant populations of stem-like Tumour Initiating Cells (TICs) and poor blood–brain barrier drug penetration. Therapies capable of effectively targeting the TIC population are in high demand. Here, we synthesize spherical diketopyrrolopyrrole-based Conjugated Polymer Nanoparticles (CPNs) with an average diameter of 109 nm. CPNs were designed to include fluorescein-conjugated Hyaluronic Acid (HA), a ligand for the CD44 receptor present on one population of TICs. We demonstrate blood–brain barrier permeability of this system and concentration and cell cycle phase-dependent selective uptake of HA-CPNs in CD44 positive GBM-patient derived cultures. Interestingly, we found that uptake alone regulated the levels and signaling activity of the CD44 receptor, decreasing stemness, invasive properties and proliferation of the CD44-TIC populations in vitro and in a patient-derived xenograft zebrafish model*.* This work proposes a novel, CPN- based, and surface moiety-driven selective way of targeting of TIC populations in brain cancer.

## Introduction

Glioblastoma, frequently abbreviated as GBM, is the most aggressive type of brain tumour with survival of less than 15 months after diagnosis^1,2^. The aggressive and therapy resistant nature of GBM is attributed in part to the characteristic intra- and inter- tumoural heterogeneity of the disease^3,4^. A plethora of genetic aberrations contribute to the unique evolution of the tumour mass in individual patients and is a consequence of the uncontrolled activation of diverse molecular pathways driving GBM invasion, progression, and therapy resistance^5^. At a cellular level, heterogeneity of GBM tumours can be described by a hierarchical model, with populations of immature Tumour Initiating Cells (TICs) feeding the growth, treatment resistance, and recurrence of GBM^6^. TICs have been extensively characterized in literature by the expression of several cell surface markers, including a trans-membrane glycoprotein receptor, CD44^7^. CD44 is activated by binding to its primary ligand Hyaluronic Acid (HA) to trigger downstream signaling via several pathways including the activation of Akt and mitogen activated protein kinase (MAPK) cascades^8^. CD44 activation results in enhanced cell proliferation and migration^9,10^, and its expression levels correlate negatively with survival times in GBM patient populations, confirmed recently in a large scale meta-analysis study^11,12^. While CD44 is not the only marker of the TIC population, it represents an attractive target to begin the design of novel targeted therapies against this aggressive initiating cell population within GBM^6^.

Developing novel therapies capable of passing the Blood–Brain Barrier (BBB) is an additional challenge required to effectively target the TIC population^13,14^. A variety of mechanisms of transport across the BBB have been described in the literature^15,16^, pointing at the limited permeability potential and call for therapeutic solutions that could overcome this limitation. The use of nanoparticles may serve as an effective therapeutic modality for GBM; however, various obstacles need to be taken into consideration, such as nanoparticle size and potential chemistry-mediated toxicity. Conjugated Polymer Nanoparticles (CPNs) are an intriguing class of nanomaterials prepared from rigid, semi-crystalline semi-conducting polymers^17–19^. Due to their remarkable optical and electronic properties, CPNs have generated a great amount of interest in recent years for the design and preparation of new theranostic agents^20^. Furthermore, previous studies have shown that CPNs have a high photothermal conversion efficiency and can be used for photoacoustic imaging^21,22^. CPNs possess many advantages over more established nanoparticles systems in theranostics and drug delivery. First, their synthesis is relatively simple, which is an undeniable advantage over other types of nanoparticles that relies on preparation at high temperature or specific self-assemblies^23^. They do not contain any metal impurities that could be toxic like their inorganic metal oxides counterparts and are stable over a wide range of temperatures, pHs and concentrations in contrast to liposomes or other micelle-like nanoparticles^24,25^. Given the unique features and characteristics of their potential benefit to anti-cancer strategies and consideration as an effective anti-glioma therapy, it is desired that CPNs exhibit a targeting specificity through design-incorporated recognition units for specific molecules on the surface of TIC populations.

Several studies across a wide range of nanomaterials provided evidence that the presence of recognition units yielded effective targeting activity and increased their anti-glioma properties^26–28^. These studies often presented highly complex designs and synthesis, thereby introducing potential obstacles for large-scale production which may impede translational applications. HA offers promise as a recognition unit when incorporated into inorganic and organic nanomaterials, providing active targeting in diverse diseases^29,30^. The role of HA in targeting specific populations of TICs in glioblastoma using CPNs has not been explored to date.

Herein, we report the design, preparation, and structural characterization of novel CPNs generated through nanoprecipitation of a diketopyrrolopyrrole (DPP)-based conjugated polymer with a fluorescein-tagged HA co-polymer (HA-CPNs). Careful and detailed evaluation using a plethora of in vitro and in vivo assays demonstrates penetration of this nano-system across the BBB, and CD44-selective and anti-proliferative effects on human glioma, mediated by the downregulation of CD44 receptor levels and its signaling activity. Despite similar design strategies being developed in other nanomaterials such as metal oxide, MRI agents or liposomes, this new design strategy does not rely on external stimuli (heat or magnetic field) to reduce tumour burden and exploits the unique versatility of conjugated polymers^8,31,32^. The obtained results confirm that these new CPNs are a strong candidate for further validation to be used in novel targeting therapies against GBM and could provide the foundation for a platform of conjugated polymer targeted therapeutics for stem cell driven cancers like GBM.

## Results

### Structural characterization of HA-CPNs

**Figure 1:**
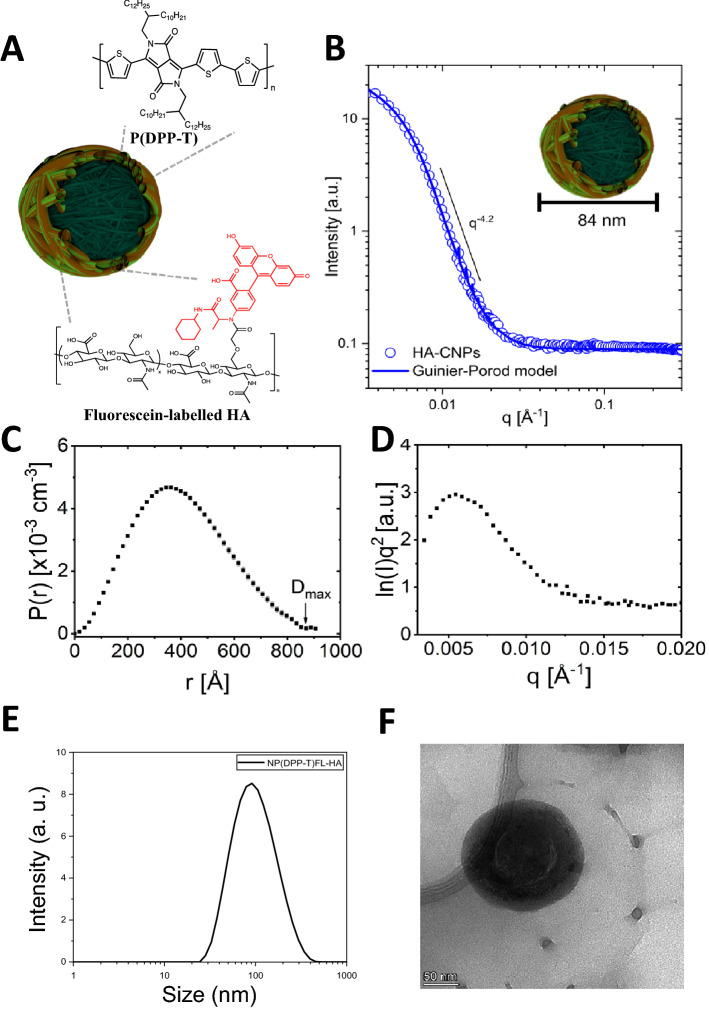
Design and Structural Characterization of the HA-CPNs. (**A**) Schematic structure of HA-CPNs; (**B**) SANS form factor for a dilute dispersion of HA-CNPs in D_2_O fit with a solid line using an empirical Guinier-Porod model. The slope in the Porod region of the plot shows a q-dependency of q^−4.2^, suggesting a spherical particle shape; (**C**) Real-space distance distribution function from an inversion approach demonstrating uniformity consistent with spherical particles of maximum dimension D_max_; (**D**) Kratky plot demonstrates a peak and plateau characteristic of compact globular structures. (**E**) Dynamic light scattering of HA-CPNs. Average diameter of 109 nm; (**F**) Transmission electron microscopy image of HA-CPNs. Scale bar of 50 nm.

In this study, a DPP-based conjugated polymer, P(DPP-T), was prepared according to previously reported literature (Figure S1, S2 and S3)^33^. In order to ensure a good solubility in tetrahydrofuran, the molecular weight of the polymer was controlled using Carother’s equation to limit the degree of polymerization. Following its synthesis, P(DPP-T) was used with commercially available fluorescein-labelled HA to prepare the HA-CPNs via nanoprecipitation (Fig. 1A). To confirm the size and to further unveil the morphological characteristics of HA-CPNs, various characterization techniques were utilized. First, the CPNs were evaluated by Small Angle Neutron Scattering (SANS). This technique, commonly used for the characterization of vesicles and lipid self-assembly, is a particularly valuable technique to probe for the shape of nanoparticles as measurements can be carried out directly in a free-floating suspension or solution without further manipulations. Scattering form factors were fit using a generalized Guinier-Porod model for an unbiased assessment of particle structure following established methodology (Fig. 1B–D)^34,35^. This analysis gives insight to the shape and size of particles based on the Porod exponent (*d*), dimensionality (*s*), and radius of gyration (*R*_*g*_). Fits to HA-CPNs yielded *d* = 4.2 ± 0.02 and s = 0.02 ± 0.06, which strongly correlates to a hard sphere with a smooth surface (*d* = 4 and *s* = 0)^34^. The extracted *R*_*g*_ = 32.6 ± 0.8 nm was extended to determine the real-space cross-sectional radius (*R* = *R*_*g*_ (5/3)^1/2^) of HA-CPNs to be 42.1 ± 1.0 nm. To confirm the results obtained by SANS, dynamic light scattering was used, with the results depicted in Fig. 1E. A polydispersity index of 0.432 with an average hydrodynamic radius (*R*_*h*_) of 54.5 nm (diameter of 109 nm) was obtained, indicative of a moderately polydisperse sample. To gain further insight on the geometry of the nanoparticles, the *R*_*g*_*/R*_*h*_ value can also be evaluated. Based on the literature, a *R*_*g*_*/R*_*h*_ ratio < 0.775 can be associated to hard spheres. Therefore, this result confirms the formation of spherical nanoparticles, as previously demonstrated for similar systems. The structure, average diameters, and spherical shape of the new CPNs were again corroborated by transmission electron microscopy as shown in Fig. 1F. CPNs showed good stability in water, 10% fetal bovine serum and phosphate buffer solutions after 5 days, confirmed by stable Zeta potential values (Table S1).

### Optimization and effects of HA-CPN uptake by glioma cells

To study the effects of HA-CPNs on glioma cell biology, it is essential to determine the optimal concentration of the particles and treatment timing allowing for the HA-CPN uptake in vitro. The U-251 MG glioma cells were treated with HA-CPNs at the concentration of 24, 48 and 96 CPNs per cell and the number of CPN-positive cells were measured at a time course up to 96 h using flow cytometry (Fig. S4a). We found the HA-CPN uptake to be concentration dependent with 95% saturation of cell population with the nanoparticles when cultures were treated with 96 particles/cell (Fig. S4a). Using HA-CPN concentrations determined not to saturate cell populations, we then measured the number of cells positive for the nanoparticles over 48 h and we found the HA-CPN’s uptake to be time dependent with significantly upregulated number of HA-CPN- positive cells at 48 and 24 h of treatment using both 24 and/or 36 particles per cell, in comparison to the 12 particles/cell incubation (Fig. S4b). Subcellular localization analysis via microscopy at 3, 24 and 48 h revealed that HA-CPNs localize primarily at the cell membrane and cytoplasm (Fig. S4c). The results allowed us to establish 24- to 48- hour cell exposure to 24 HA-CPNs/cell as optimal parameters to be applied and tested in further experiments.

### HA-CPN uptake is cell cycle phase dependent

It has been demonstrated previously that the efficiency of the nanoparticle uptake can be dependent on the phase that cells are in within the cell cycle^36^. To determine the impact of cell cycle phase on the uptake of tested nanoparticles, U-251 MG cells were synchronized in select cell cycle phases. HA-CPNs at 24 particles per cell, were introduced for 3 h into the synchronous cultures and the uptake was analyzed using flowcytometry. Enrichment in G0/G1 was accomplished using double thymidine block. Starvation (0% FBS, 24 h) resulted in approximately 70% of cells to reside in G1/G0 and over 11% and 16% in S and G2/M phases, respectively. The enrichment in G2/M phase was accomplished upon the treatment with nocodazole (2 pg/mL) (Fig. S4e). We found that mitotic populations of cells had the highest number of cells positive for HA-CPNs, when compared to starvation and thymidine treated cultures, accounting for 70% of the population tested (Fig. S4f.).

### HA-CPNs demonstrate selective uptake and anti-proliferative properties in vitro

**Figure 2:**
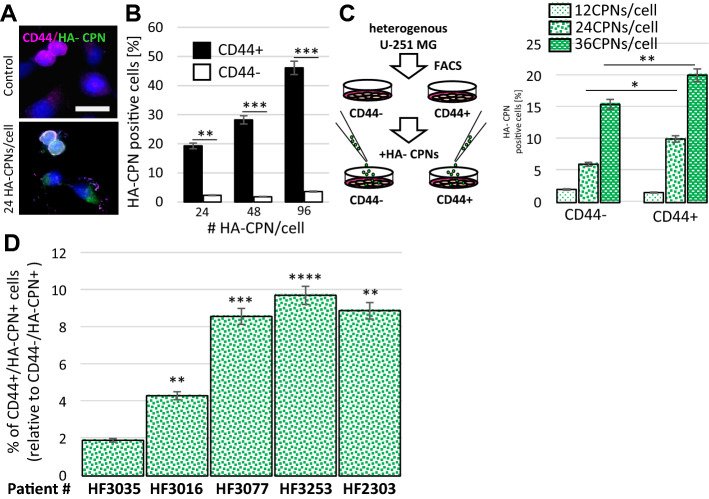
HA-CPNs Demonstrate Selective Uptake by CD44 Enriched U-251 MG Cells as well as GBM Patient-derived Cells. (**A**) Representative image of cells stained with anti-CD44 antibody and treated with 24 HA-CPNs/cell or vehicle control (Control); scale bar: 25 μm; (**B**) Flowcytometry analysis of U-251 MG cells stained with anti-CD44 antibody and treated with 24, 48, 96 HA-CPNs/cell or vehicle control (Control). Cells positive for HA-CPNs graphed as % of cells positive (CD44 +) and/or negative (CD44-) for the antibody stain; (**C**) Flowcytometry analysis of HA- CPN positive U-251 MG cells in FACS- derived CD44 + and CD44- populations treated with 12, 24, 36 HA-CPNs/cell graphed as % of the population tested; (**D**) GBM patient- derived cultures (HF3035, HF3016, HF3077, HF3253, HF2303) tested for CD44-selective HA-CPN uptake and quantified as % of CD44 + cells relative to the % uptake in CD44- cells. Data shown as mean ± s.d, n = 3, *p < 0.05, **p < 0.01, ***p < 0.001; Student’s *t*-test.

To determine whether the HA molecules, present on CPNs, mediate uptake of the nanoparticles in glioma cells in vitro, U-251 MG cells were incubated with HA-CPNs, and conjugated polymer nanoparticles prepared from polysorbate that do not contain HA (Nc-CPNs) as control (see Supplementary Information for details). CPNs possess weak innate fluorescence (524 nm) which allowed for signal quantification and comparative analysis in this experiment using same excitation/emission spectrum for both treatments. The cells were treated with 24 CPNs per cell for 1 h, washed, and the innate fluorescent signal was quantified at the indicated time points using a plate reader. We found an increase in fluorescence at 0.5-h and 1-h timepoint when cells were treated with HA-CPNs in comparison to Nc-CPN treatment at the same timepoints (Fig. S4d). To address the potential selectivity of HA-CPNs towards glioma populations characterized by CD44 expression, U-251 MG cells were incubated with increasing concentrations of HA-CPNs for 48 h followed by staining with a fluorescent anti-CD44 antibody. The HA-CPN uptake and co-localization with CD44 was monitored using microscopy (Fig. 2A). We found co-localization to occur at the cell membrane and noted HA-CPN uptake in the cytoplasm (Fig. 2A). The number of HA-CPN positive cells in relation to CD44 expression was assessed using flowcytometry (Fig. 2B). At 24, 48, and 96 HA-CPNs/cell tested, there was significantly higher uptake (~ 8, ~ 16 and ~ 13-fold, respectively) of the HA-CPN by populations staining positively for CD44 in comparison to CD44 negative cells with (Fig. 2B). Compartmentalized, 24-h exposure to HA-CPNs by separate, FACS- derived CD44 + and/or CD44- U-251 MG cells (Fig. 2C, left) revealed that although separated populations of CD44- cells were characterized by non-specific uptake of the nanoparticles (Fig. 2C, right), CD44 + cells demonstrated significantly increased enrichment for HA-CPNs in comparison to CD44- cells, at 24 and 36 HA-CPNs/cell, showing the rate of uptake of 10% per every 24 h, at 24 HA-CPNs/cell (Fig. 2C, right), which was consistent with treatment of heterogeneous U-251 MG populations (Fig. 2B).

Highly propagated and commercially-available cell lines fail to mimic the actual state of the disease and may produce false results in functional assays and therapy response screens, in vitro and in vivo*.* To further examine the selectivity of the nanomaterials system, using life- relevant model, we employed cell cultures derived from tumours obtained at the time of surgery, from individual GBM patients. Five GBM patient- derived cultures were treated with HA-CPNs for 7 days, stained with CD44 antibody and the uptake was analyzed via flowcytometry. All five cultures showed an increase in CPN uptake in the CD44 + population as compared to the CD44- population, with the % uptake being statistically significant in four out of five of the cultures (Fig. 2D). This data supports the potential for using nanoparticles selective in targeting TIC populations in glioma.

### Proliferation and stemness of primary GBM patient derived cells are decreased by HA-CPN treatment

**Figure 3:**
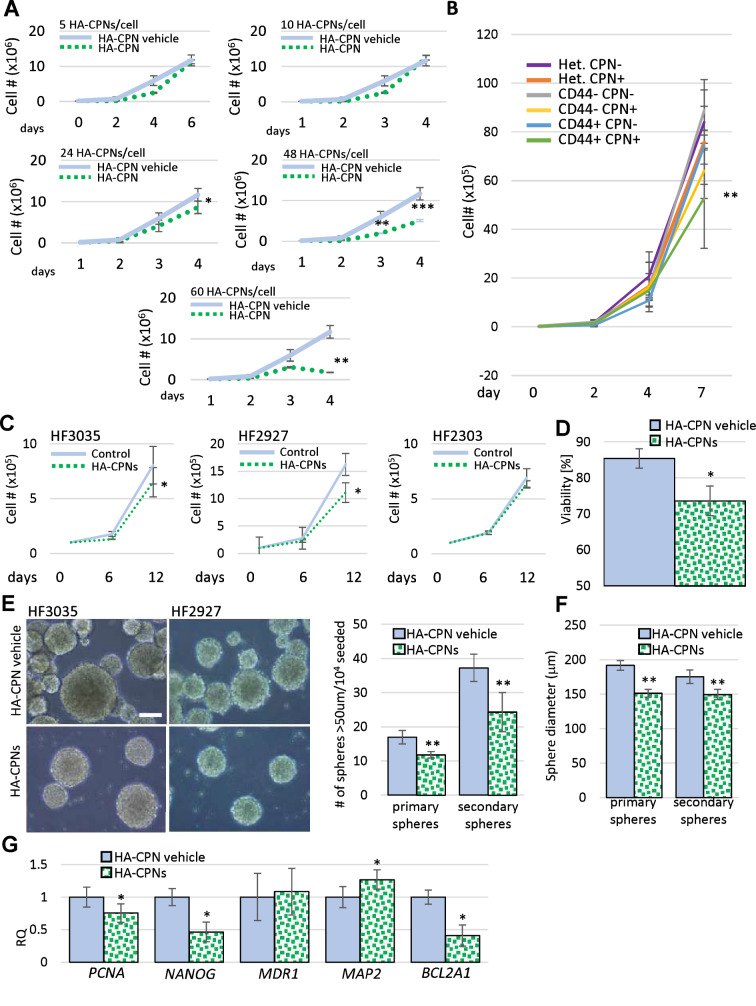
Effects of HA-CPNs on Proliferation and Stemness of Primary GBM Patient- derived cell lines. (**A**) U-251 MG cells treated with indicated concentrations of HA-CPNs or vehicle control (HA-CPN vehicle) and subjected to trypan blue exclusion assay over a time course of 6 days; (**B**) Proliferation of heterogeneous (Het.), CD44 + and CD44- populations of U-251 MG cells, FACS- enriched for HA-CPNs (HA-CPN +) compared to HA-CPN- cells, assessed using trypan blue exclusion assay; (**C–G**) Patient lines, HF3035, HF2927 and HF2303, treated with 24 HA-CPNs/ cell and/or HA-CPN vehicle control and subjected to: a viability assay- average number of viable cells assessed over three cell lines tested, quantified as % of total population via trypan blue exclusion assay (**C**), a proliferation assay over the time course of 12 days- cell number assessed via trypan blue exclusion assay at the indicated time points (**D**), neurosphere formation assay, representative images of HF3035 and HF2927 (left); scale bar = 100 μm. Number of spheres formed is quantified per every 10^4^ cells seeded, three lines averaged (**E**), assessment of an average diameter of spheres formed; three lines pooled for analysis (**F**), qRT-PCR analysis of mRNA levels of the indicated markers of stemness/differentiation (**G**). Data shown as mean ± s.d, n = 3, *p < 0.05, **p < 0.01; Student’s *t*-test.

To test the effects of HA-CPNs on glioma proliferation, we treated U-251 MG cells with 5, 10, 24, 48, 60 HA-CPNs/cell over a time course of 6 days. The concentrations tested, up to ~ 24 HA-CPNs/cell, did not affect cell proliferation (Fig. 3A). A statistically significant decrease of the cell number was found at the concentrations of 48 and 60 HA-CPNs/cell, when compared to the vehicle control (Fig. 3A). To assess whether the exposure of glioma cells to HA-CPNs influences GBM metabolic activity in vitro*,* we subjected U-251 MG cell line to MTT assay in the presence of the same particles/cell concentration range as for the proliferation assay. A significant decrease in metabolic activity relative to the vehicle control at 72 h, was found for cultures treated with 24, 48, and 60 HA-CPNs/cell (Fig. S5). The results demonstrated that the exposure to HA-CPNs results in cytostatic effects in a concentration and time- dependent manner. To determine whether selective uptake of HA-CPNs by CD44 + glioma populations can impact their proliferative potential CD44-, CD44 + and heterogeneous populations of U-251 cells were treated with HA-CPNs for 7 days and sorted based on the HA-CPN content and proliferation was monitored over 7 days. CD44 + cells containing CPNs were found to have a significant reduction in proliferation over all other cell populations (Fig. 3B).

To test the impact of HA-CPNs on glioma proliferative potential in cells derived from GBM tumours, primary cultures (HF3035, HF2927, HF2303) were treated with HA-CPNs along with the vehicle control. Trypan blue exclusion assay demonstrated an average decrease of 15% in viability of the tested lines (Fig. 3C). A significant decrease of proliferation was observed in two, out of three patient- derived cultures treated with 24 HA-CPNs/cell and/or vehicle control (Fig. 3D). At 12 day- timepoint, the proliferation of HF3035 and HF2927 was inhibited by 19.3% and 31.5%, respectively, when incubated with HA-CPNs as compared to the vehicle control. This could be attributed to the observed average decrease of viability in those lines (Fig. 3D). Both lines demonstrated an average of 22% decrease of proliferation at 6 h which was consistent with observed decreased proliferation in U-251 MG cells treated with 24 HA-CPNs/cell for the same period of time (Fig. 3A). Given that HA-CPNs selectively target CD44 + TICs, neurosphere formation assay was performed to determine if the nanoparticle treatment can regulate stemness in GBM patient derived cell lines. We showed that primary GBM cells treated with HA-CPNs produced significantly lower number of spheres in two consecutive generations of spheres (primary and secondary) (Fig. 3E) and the generated spheres were of smaller diameter (Fig. 3F). Consequently, qRT-PCR analysis of well- established stemness markers showed significantly downregulated mRNA expression levels of *PCNA*, *NANOG* and upregulated levels of *MAP2*, differentiation marker, in patient derived cultures, treated with HA-CPNs in comparison to the vehicle control (Fig. 3G). We also observed significant decrease of BCL2A1, an anti-apoptotic marker. The mRNA levels of MDR1, a crucial TIC- related drug resistance marker, remained unaffected by the HA-CPN treatment (Fig. 3G). The obtained data show that HA-CPNs not only negatively regulate proliferation of glioma in primary patient cultures but also decrease stemness of GBM cells, suggesting that treatment with HA-CPNs can better the treatment, not only as a potential therapy “carrier” via selective binding of TICs but also through direct regulation of TIC aggressiveness.

### Migration and invasion of GBM is negatively regulated in HA-CPN treated cultures

**Figure 4:**
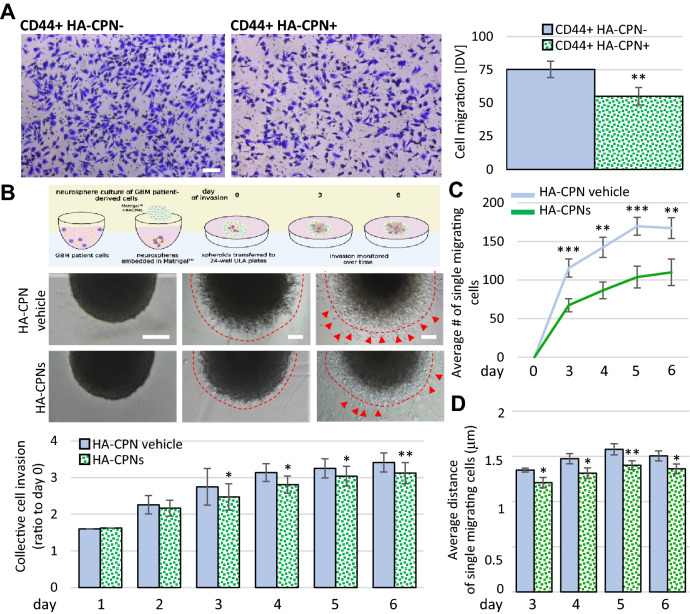
Treatment with HA-CPNs Regulates Migration and Invasion in Glioma. (**A**) Boyden chamber assay using CD44 + U-251 MG cells treated and FACS- enriched for HA-CPNs (CD44 + HA-CPN +) compared to HA-CPN- cells (CD44 + HA-CPN-). Representative images (left). Scale bar = 100 μm. Migrated cells scored in five fields of view per replicate over three replicates, using ImageJ as Integrated Density Value (IDV) (right); (**B, C**) Matrigel™ invasion assay using GBM patient derived spheres treated with HA-CPNs and/or HA-CPN vehicle control over a time course of six days. Schema of the protocol (top), representative images (mid panel). Scale bars = 100 μm; (**B**) Schema representing sphere invasion assay in Matrigel^TM^; Created with BioRender.com (top) and collective invasion measured using ImageJ as change in sphere radius over time. Quantified values averaged over 6 spheres per treatment, per time point, over 3 patient cell lines (bottom). Leading edge assessed optically, marked by dashed line; (**C**) Average number of single cells migrating (red arrowheads in **B**) scored using ImageJ per each Matrigel™- embedded sphere at the indicated timepoints; (**D**) Average distance of the single cell migration at the indicated timepoints. Data shown as mean ± s.d, n = 3, *p < 0.05, **p < 0.01, ***p < 0.001; Student’s *t*-test.

To further investigate the influence of the HA-CPNs on glioma aggressiveness, we analyzed the migratory and invasive properties of patient-derived GBM cells treated with the nano-system. CD44 positive cells enriched for HA-CPNs via FACS (HA-CPN +) were used in parallel to FACS- derived CD44 + cells negative for HA-CPN uptake (HA-CPN-). Boyden chamber assay showed that HA-CPN + cells migrated significantly less in comparison to HA-CPN- cells (Fig. 4A). We also tested the effects of the HA-CPNs on invasion of primary patient cells over a time course (Fig. 4B). Using single spheres generated from primary patient cells embedded in Matrigel™, supplemented with either vehicle control or 24 HA-CPNs/cell we demonstrate that presence of HA-CPNs in the Matrigel significantly decreased the distance of invasion compared to the vehicle control. The analysis of invading single cells revealed that significantly more cells detached from the spheres when Matrigel™ was supplemented with the vehicle control as compared to HA-CPN- supplemented Matrigel™ (Fig. 4B, top and Fig. 4C). In comparison to the vehicle control, single cells travelled a shorter distance of invasion in the presence of HA-CPNs (Fig. 4D). The demonstrated data support that HA-CPNs play a role in suppressing major hallmarks of GBM aggressiveness when tested on primary cells in vitro.

### Uptake of HA-CPNs is regulated by Clathrin Mediated Endocytosis (CME) and decreases expression and activity of CD44

**Figure 5:**
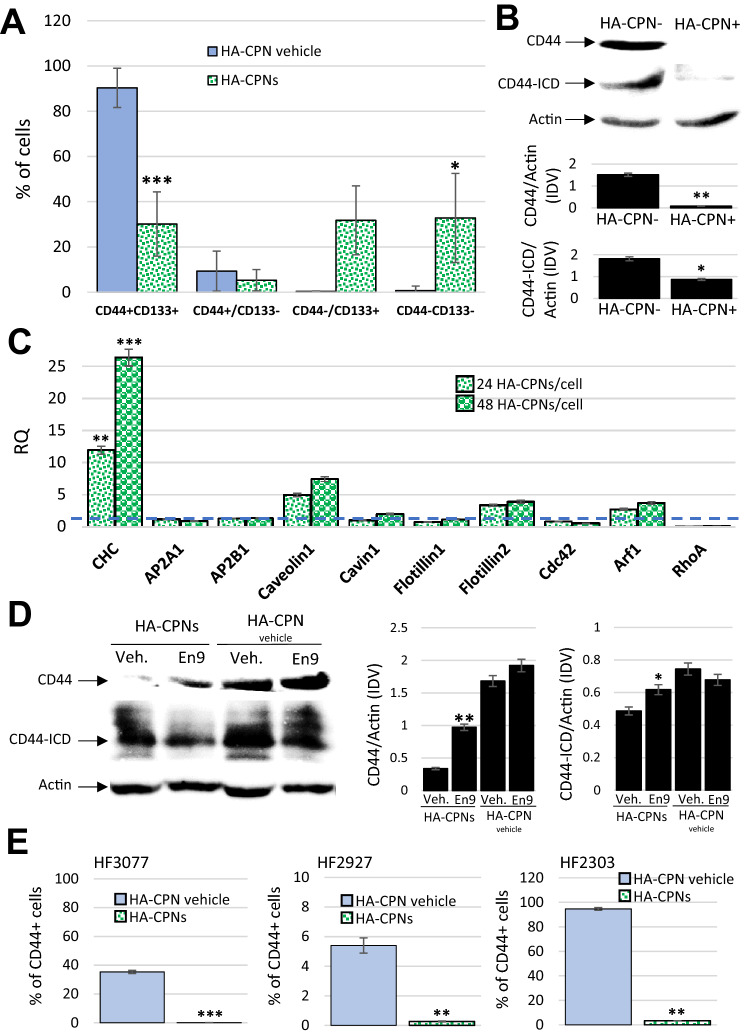
HA-CPNs Downregulate the Expression Levels and Signaling Activity of CD44 in GBM. (**A**) U-251 MG cells were treated with HA-CPNs and/or vehicle control (HA-CPN vehicle) and subjected to flowcytometry analysis of the indicated TIC populations; (**B**) Assessment of the protein expression levels of CD44 and CD44 intracytoplasmic domain (CD44-ICD) via Western blotting in FACS derived HA-CPN + and HA-CPN- cells (top). Protein levels quantified using densitometry and shown as ratio to Actin in Integrated Density Values (IDV) (bottom, graphs); (**C**) mRNA expression of signature markers of diverse endocytotic mechanisms in U-251 MG cells treated with 24 and/or 48 HA-CPNs/cell relative to HA-CPN vehicle control (dotted line); (**D**) CD44 and CD44-ICD protein expression levels in U-251 MG cells treated with HA-CPNs and/or HA-CPN vehicle control in the presence of Endosidin 9 or vehicle control (left). Protein levels quantified using densitometry and shown as ratio to Actin in Integrated Density Values (IDV) (right); (**E**) Flowcytometry analysis of CD44 expression levels in the indicated GBM patient samples treated with HA-CPNs and/or HA-CPN vehicle control. Data shown as mean ± s.d, n = 3, *p < 0.05, **p < 0.01, ***p < 0.001; Student’s *t*-test. Original blotted membranes of Western can be found in Fig. S7.

To assess the impact of HA-CPN treatment on GBM TIC content we analysed the composition of TIC populations in heterogeneous samples of U-251 MG cells using antibodies against CD44 and CD133. CD133 is an established indicator of stemness in glioblastoma and its expression with CD44 has been demonstrated previously to drive expansion of TICs^37,38^. Flowcytometry analysis revealed that treatment with HA-CPNs causes significant downregulation of glioma cells positive for both CD44 and CD133 markers (CD44 + , CD133 +) leading to overall decrease of TICs in the studied population with significant increase of cells negative for either of the TIC markers (CD44-, CD133-) (Fig. 5A). CD44 protein expression levels were then assessed in lysates from U-251 MG cells sorted for the HA-CPN content (HA-CPN + vs. HA-CPN-). We found that CD44 expression levels were significantly downregulated in HA-CPN + as compared to HA-CPN- cells (Fig. 5B). It has been established previously that upon sequential proteolytic cleavage CD44 can produce CD44 intracytoplasmic domain (ICD) tail which translocates to the nucleus, and is involved in transcription of genes responsible for tumor invasion and therapy resistance^39–41^. Our protein expression analysis shows that the HA-CPN- enriched U-251 MG cells have significantly reduced levels of CD44-ICD in comparison to HA-CPN negative cells (Fig. 5B). To assess the endocytotic mechanism responsible for HA-CPN uptake we established an expression profile of several markers of endocytosis in U251 MG cells treated with increasing concentrations of HA-CPNs (24 and 48 HA-CPNs per cell) in comparison to the vehicle control. Using both concentrations of HA-CPNs we found significantly upregulated mRNA expression levels of Clathrin Heavy Chain (CHC) which is indicative of upregulation of CME (Fig. 5C)^42^. Analysis of protein expression levels in U251 cells treated with HA-CPNs in the presence or absence of CME inhibitor, Endosidin9, not only confirmed the previously observed significant decrease of CD44 and CD44-ICD levels in cells treated with HA-CPNs in comparison to HA-CPN vehicle control but also demonstrated the decrease was rescued in cells treated with Endosidin9 (Fig. 5D), suggesting that CME plays an important role in the HA-CPN mediated effects in glioblastoma. To determine whether the observed HA-CPN- mediated regulation of CD44 receptor levels can affect TIC populations in glioblastoma patients, TICs derived from three individual GBM patient samples were treated with the nanoparticles and/or vehicle control. Flowcytometry analysis showed a significant decrease of the level of CD44 + cells in patient samples treated with HA-CPNs in comparison to the vehicle control, in all GBM cultures tested (Fig. 5E). The results suggest that observed effects of HA-CPNs in glioma might be due to decrease of the levels and pathway activity of CD44 receptor.

### HA-CPNs demonstrate selectivity for CD44+ glioma cells in vivo and decrease tumour burden in patient-derived xenograft (PDX) zebrafish models of human GBM

The inability to penetrate the BBB by therapeutic agents is one of the biggest obstacles in an effective and lasting treatment of GBM. Hence, an effective therapy of GBM via systemic administration of HA-CPNs is dependent their ability to efficiently cross the BBB. To assess whether the BBB is permeable to HA-CPNs, we injected the nanoparticles into the yolk sac of zebrafish embryos at 48 h post fertilization (hpf) and analyzed their biodistribution over a period of two weeks. We found that at 5 h post injection (hpi) HA-CPNs entered the duct of Cuvier inside the yolk sac and became clearly detectable with increasing levels in the head/brain area at 8hpi and 24hpi (Fig. S6a). HA-CPNs were still present at quantifiable levels at 24hpi and 14 days post injection (dpi) (Fig. S6a).

**Figure 6:**
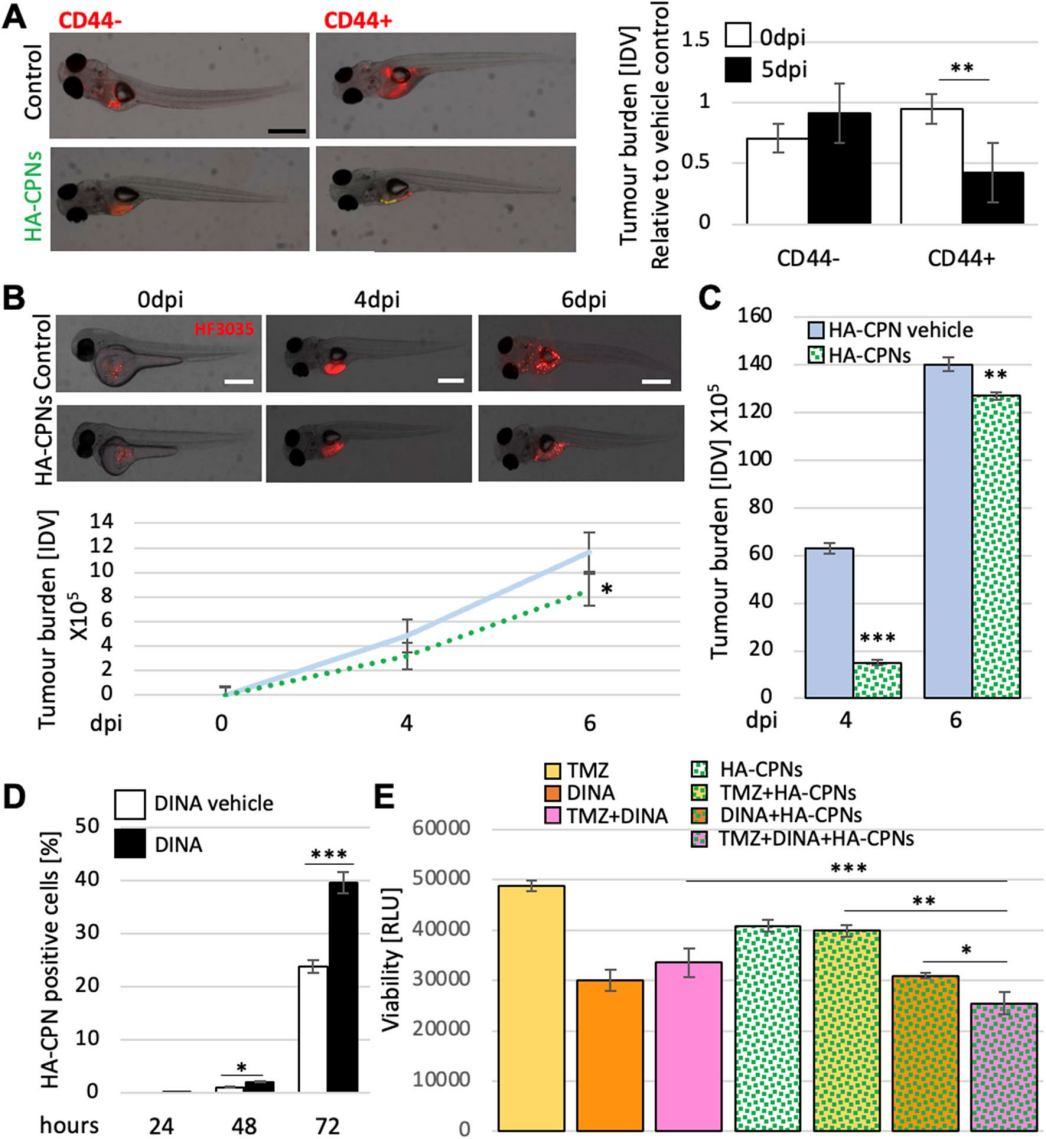
Decrease of Tumour Burden in GBM Zebrafish PDXs and Enhanced Treatment Sensitivity upon Exposure to HA-CPNs. (**A**) Representative image (left) and tumour foci burden analysis (right) in zebrafish embryos injected with fluorescently labelled (red) CD44 + and/or CD44- cells and analysed at 0- and 5-days post injection. Tumour burden quantified and graphed as Integrated Density Values (IDV) for HA- CPN treatment/ HA-CPN vehicle control treatment (Control) at the indicated time points; (**B**) HF3035 and HF2303 GBM patient-derived zebrafish PDX models treated with HA-CPNs compared to HA-CPN vehicle control; representative images of PDX using HF3035 (top) and average tumour burden quantified using ImageJ as IDV at the indicated timepoints (bottom) over two GBM patient lines; (**C**) Average tumour burden quantification at the indicated timepoints in PDXs derived from CD44 + HF3035 and CD44 + HF2303 GBM lines treated with HA-CPNs compared to HA-CPN vehicle control (Control) over time; (**D**) Cells treated with dinaciclib and/or vehicle control (Control) in the presence of 24 HA-CPNs/cell. Nanoparticle uptake analysed using flowcytometry at the indicated time points; (**E**) Cell viability quantified using a luminescent assay at 72 h post HA-CPN and drug treatment, as monotherapy or in combination as indicated (TMZ, Temozolomide; DINA, dinaciclib). Data shown as mean ± s.d, n = 3, *p < 0.05, **p < 0.01, ***p < 0.001; Student’s *t*-test.

To investigate the effects and selectivity of HA-CPNs in glioma proliferation in vivo, zebrafish embryos at 48hpf were injected with either FACS- derived CD44 + and/or CD44- or heterogeneous U-251 MG cells or patient derived cell lines in the presence of HA-CPNs and/or vehicle control and analyzed up to 6dpi populations (Fig. S6b). To investigate the tumour burden kinetics in the presence of the nanoparticles in comparison to the vehicle control, 2dpf zebrafish embryos were injected with the co-suspension of CD44 + and CD44- U-251 MG cells labeled with different fluorescent cell trackers in the presence of HA-CPNs or their absence (vehicle control) (Fig. S6C, left). Tumour foci formation was monitored over time and images were taken at the indicated time points (Fig. S6c, left). We found that the extent of tumour burden from co-injected populations in the presence of HA-CPNs was significantly decreased for CD44 + derived foci but not for CD44- ones at 24hpi and 4dpi of the time course (Fig. S6c, right). CD44 + and CD44- U-251 MG cells were then injected separately into the zebrafish in the presence of HA-CPNs and/or vehicle control, and at least fifty embryos per treatment were imaged at 5dpi (Fig. 6A) and subjected to fluorescent signal quantification (Fig. 6A, right). Comparative analysis revealed a significant decrease of the CD44 + but not CD44- foci burden at day 5 of the nanoparticle treatment when compared to U-251 MG cell burden at injection (0dpi) (Fig. 6A, right).

To further validate anti-glioma properties of HA-CPNs in vivo, we generated zebrafish PDX models using two individual GBM patient-derived cultures. Both, heterogeneous populations of cells and/or FACS sorted CD44- positive and negative cells were employed. Consistently with U-251 MG in vivo data, we found that in comparison to the vehicle control, HA-CPNs were significantly decreased overall tumour burden in zebrafish PDX models established using heterogenous patient lines, over a time course of 6 days post injection (Fig. 6B). It is notable that zebrafish PDX models derived using CD44 enriched cell populations increased tumour burden in both the vehicle control as well as HA-CPNs, and over a 6-day time course the effects of CPNs were reduced in efficacy at later time points (Fig. 6C). This suggested the potential need to test combination of HA-CPNs with other therapeutics to improve the treatment response. Unpublished data from our lab revealed that exposure of U251 MG cells to 15 nM CDK inhibitor, dinaciclib, enriches the culture for CD44 + cells (not shown). To assess if dinaciclib could aid in selectivity of HA-CPNs in glioma in vitro, the nanoparticle uptake was assessed in U-251 MG cells treated with dinaciclib in comparison to the vehicle control. At the 72-h timepoint there was a significant increase in the number of HA-CPN positive cells in both the drug treatment and the control, as compared to the earlier 24- and 48- hour time points; however, the dinaciclib treated cells demonstrated significantly higher uptake of HA-CPNs than vehicle treated cultures at 48 and 72 h (Fig. 6D). In vitro drug screening assay conducted using three patient derived cultures (HF3035, HF2927, HF2303) demonstrated a significant decrease in viability when cells were treated with standard of care Temozolomide (TMZ) in triple combination with dinaciclib (DINA) and HA-CPNs, in comparison to TMZ alone or dual treatment with TMZ + dinaciclib, TMZ + HA-CPNs and/or dinaciclib + HA-CPNs, in three lines combined (Fig. 6E). This data suggests a potential therapeutic benefit from applying HA-CPNs in combination with known therapeutics against glioma especially in tumours resistant to the available standard of care treatment.

## Discussion

Desperate search for novel effective therapeutics against the deadliest forms of cancer has integrated several distinct disciplines in recent years. Materials chemistry is a fast-growing field of chemical research which, by offering avant-garde treatment concepts, changes the mindset of how the disruptive action against cancer cells can be executed. Eradication of extremely heterogeneous and dynamic types of cancer such as GBM calls for multimodal targeting strategies. To the best of our knowledge, this study is the first one to demonstrate the application of conjugated polymers in the generation of nanoparticle system presenting tumour initiating cell- selective properties in human glioblastoma. Previously reported nano-systems such as PEG- based micelles, liposomes or magnetic nanoparticles have been functionalized to target CD44 positive cancer cells, however, none of those studies investigated role of the proposed approaches in targeting glioblastoma CD44 + tumour initiating cells and required BBB penetration^43–45^. CPNs are prepared to target CD44 positive cells via hyaluronic acid, as peripheral recognition moieties, and although the incorporation of HA in nano-based systems has been exploited in previous literature on drug delivery, this novel conjugated polymer-based system offers several advantages over other inorganic and organic nanoparticles^46^. We show here that novel utilization of this chemistry, offering purity, stability, low toxicity, and BBB penetration, in a format of a selective nanoparticle allows for efficient targeting of aggressive cell populations in human glioma in vitro and in vivo*.*

The HA-CPNs were structurally characterized using advanced characterization techniques for nanoparticles, including small-angle neutron scattering, dynamic light scattering, and electron microscopy. We successfully confirmed that the HA-CPNs are spherical with an average diameter of 109 nm and have a relatively uniform size distribution as confirmed by low dispersity index. To get a good overview of the new platform developed, comparison with previously reported nanomaterials was made and the results are depicted in Table 1. In comparison to other nanomaterial-based systems, the new CPNs possess some advantages, particularly with regards to their potential to be transported across the BBB and intrinsic toxicity against GBM. Furthermore, in contrast to other types of nanomaterials that often require external treatment (such as extrusion) to control sizes and shapes, the fine-tuning and facile control over the structural parameters of the CPNs is a favorable feature to maximizes their potency as theranostic agents to be transitioned across the BBB^47,48^.

**Table 1:** Comparison of the new HA-CPNs with selected previously reported nanomaterials against GBM.

Materials	External stimuli	Selectivity	Evidence of transport across BBB	Demonstrated intrinsic toxicity to GBM in vivo	Evaluated in primary patient cultures	Refs
HA-MnO_2_ nanoparticles	Magnetic field	Yes	No	No	No	^32^
HA liposomes	None	Yes	Yes	Yes	No	^31^
HA lipid nanoparticles	None	Yes	Yes	No	No	^8^
DPP-based CPNs	Laser irradiation	No	No	No	No	^49^
HA-CPNs (this work)	None	Yes	Yes	Yes	Yes	–

This study optimized biological application of HA-CPNs using human glioma U-251 MG cell line and validated the behavior of HA-CPNs in an in vitro and in vivo biologically relevant setting using GBM cultures obtained at surgery from individual GBM patients. Assessment of the incubation time, along with the concentration of nanoparticles are essential to properly devise the parameters to the biological system and allowed us to standardize the concentration used across several experiments. We found HA-CPN uptake to be dependent on both time and CPN concentration. Nanoparticle uptake heavily depends on several changes occurring at the cell membrane including but not limited to electrostatic interactions between nanoparticle and the membrane^50^, hydrophobic and interfacial forces^51,52^, as well as the type and efficiency of the endocytotic mechanism responsible for the internalization of the nanoparticles^53^. Hence, uptake of nanoparticles can fluctuate in diverse growth settings^54,55^. We demonstrate that HA-CPN uptake varies at different phases of the cell cycle, and that cells permitted to undergo cell cycle progression through G1-S-G2 were able to accumulate the highest number of HA-CPNs. This may support that HA-CPNs will accumulate in more rapidly dividing tumourigenic cell populations. Our results are with other studies that have shown that different cell cycle phases internalize nanoparticles at different rates with the highest uptake occurring at G2/M, followed by S, and then G0/G1 phase^36,56^.

Importantly, this study explored the specificity of HA-CPNs in targeting glioma initiating cells which are positive for CD44 receptor. To determine whether the CD44 ligand, HA, present on CPNs plays a role in the nanoparticle selectivity, the U-251 MG cells and/or patient primary lines were sorted via FACS to obtain CD44 + and CD44- cell populations. Alternatively, heterogeneous population of U-251 MG cells was treated with HA-CPNs and then stained with a fluorescent antibody and co-expression of both signals was measured by flowcytometry. Both approaches demonstrated a significant increase of uptake in CD44 + cells as compared to CD44- cell populations by U-251 MG cells as well in four out of five primary GBM cultures tested.

Several studies have shown that HA–receptor interaction promotes glioma proliferation and invasiveness, and abrogation of the interaction leads to diminished glioma aggressiveness^57–59^. Hence it was important to determine if HA-CPNs would trigger any biological activation of the CD44 receptor. Surprisingly, we show that treatment with increasing concentrations of HA-CPNs for prolonged time leads to a downregulation of proliferation, metabolic activity and stemness in GBM-patient derived cultures. We show that exposure to HA-CPNs decreases migration in collective as well as single cell invasion of GBM primary cultures. Our results support that the HA-CPNs decrease CD44 signaling activity by downregulating the receptor protein levels as well as the protein expression levels of the product of sequential CD44 cleavage of the intracytoplasmic domain (CD44-ICD). The role of CD44-ICD as the signal transduction molecule has been explored in diverse types of cancer including glioma and its role in initiating transcription, promoting stemness and migration/invasion, processes explored in this paper, has been well established over the years^41,60,61^. Dissecting the details of the mechanism and downstream molecular effects of the HA-CPN mediated regulation of CD44-ICD is of high priority and warrants further investigation.

In vivo experiments to address the biological effects of HA-CPNs using zebrafish revealed penetration of the nanoparticles across the BBB over time. Although the developmental and molecular mechanisms of the functional BBB of zebrafish remain under intensive investigation by many^62–64^, it has been demonstrated that character of cerebral microvessels and the properties of BBB in the zebrafish embryo are similar to mammals^64,65^. Hence, zebrafish has been utilized previously to investigate and model BBB penetration by nanoparticles of future therapeutic potential. Our results show BBB crossing and distribution of portions of HA-CPNs to the brain which is consistent with previously published results using a different type of a nanoparticle^62^. The mechanisms regulating the characteristics of biodistribution of HA-CPNs over time, including, the level of the nanoparticle containment in the vascular compartment and the level of the diffusion within brain tissue requires careful investigation followed by validation using mammalian in vivo models to further characterize the process by which HA-CPNs reach the tumour site within the brain. Using zebrafish model, we show that HA-CPNs cause selective growth inhibition of the CD44 + but not CD44- tumour foci over time, either in individual xenografts or using CD44 + /CD44- co-injection model. Consequently, using zebrafish PDX model we demonstrate that targeting of the CD44 + populations in vivo results in significantly abrogated growth of tumour bulk supporting the efficacy of therapeutic strategies targeting TICs in glioma. Providing treatment solutions for tumours presenting therapy resistance is of highest demand. Our results in vitro show that combining standard of care TMZ therapy with an agent selective for CD44 cell populations and with HA-CPN treatment successfully abrogates viability of TMZ- resistant GBM cells validating this nanoparticle system for further testing in pre-clinical models.

## Conclusion

Expression of the cell surface receptor CD44 has been correlated with GBM aggressiveness and poor patient prognosis^11,66^, suggesting that targeting of CD44 + populations may have significant effects on clinical outcomes. A careful, multi-assay evaluation provided in this study demonstrates that DPP-CPNs, generated with HA, can selectively target CD44 + cells in tested GBM patient- derived cultures. Interestingly, this work provides the first evidence that this form of targeting may alone exert anti-tumourigenic properties in vivo*,* a mechanism that may be driven by downregulating the protein expression and signaling levels of CD44. The versatility of the CPN system also allows for future modification and elaboration of personalized approaches. We propose that this system represents a novel targeting therapy approach against aggressive stem-cell driven cancers such as glioblastoma.

## Methods

Details on P(DPP-T) synthesis and the instruments used for its characterization can be found in the Supplementary Information. The experiments were approved by the University of Windsor’s Animal Care Committee (ACC). All methods were performed in accordance with the relevant guidelines and regulations, including with ARRIVE guidelines.

### Nanoprecipitation

1 mg of P(DPP-T) conjugated polymer and 2 mg of the fluorescein hyaluronic acid surfactant were dissolved in 1 mL of tetrahydofuran (THF) and left to stir for 30 min. The solution was then injected in 6 mL of deionized water under probe sonication (amplitude of 60, power of 55 W for 5 min). After sonication, the sample was passed through a 0.2 µm PTFE (hydrophobic) syringe filter to remove any large aggregated materials. The nanoparticles (97% yield as determined by drying under ultra-high vacuum) were washed multiple times with deionized water and the resulting aqueous suspension was stored at 4 °C prior to use. The vehicle control and Polysorbate-containing conjugated polymer nanoparticles (Nc-CPN) were prepared via analogous protocols.

### Cell culture and HA-CPN treatment

U-251 MG wt cells were obtained from (Dr. Rutka, SickKids Hospital, Toronto). The cells were cultured at 37 °C and 5% CO_2_ in Minimum Essential Medium Eagle (EMEM), (Sigma-Aldrich) supplemented with 10% Fetal Bovine Serum (FBS) (Gibco, #10,437,028), 1% Penicillin and Streptomycin (Invitrogen, #15,140,148), 1 mM sodium pyruvate, and with 1 × non-essential amino acids (NEAA) (Sigma, #M7145). A multi-laser nanoparticle tracking analysis system (Horiba ViewSizer 3000) was used to determine the nanoparticle concentration at 1 mL. CPNs were added directly to the media in volume corresponding to the desired number of CPNs/cell concentration, THF-evaporated water was used as vehicle control in corresponding volumes. For the proliferation assay the concentration of CPNs/cell was varied and included 5, 10, 24, 37, and 53 CPNs/cell (10μL, 20μL, 45μL, 70μL, and 100μL of CPNs or the vehicle control) per 2 mL of growth media. For the MTT assay the applied concentrations of CPNs/cell included 6, 12, 24, 48, and 63 CPNs/cell (3μL, 7.5μL, 15μL, 30μL, and 45μL of CPN solution or vehicle control). The cultures treated with CPNs were protected from light. For immunocytochemistry, cells were cultured on coverslips for 2, 4, 7, and 24-h with 24 CPNs/cell.

### GBM patient-derived cultures

Resected tumors were collected at the Henry Ford Hospital, with written consent from patients in accordance with institutional guidelines as approved by the Institutional Review Board at Henry Ford Hospital. Pathology was graded according to the WHO criteria. The cells were extracted and cultured as spheres as described previously^66^.

### Cell cycle synchronization

Double thymidine block was performed using 2 mM thymidine for 18 h. At the 18-h mark, the media was replaced with regular growth media and incubated for 9-h before thymidine was re-added to the plates for another 18-h incubation. Cell starvation included 24-h incubation of cells in media supplemented with 1% FBS. Mitotic population enrichment was achieved by incubation of cultures with 2 pg/mL nocodazole for 24 h.

### Flow cytometry

Single cell suspension (10^6^ cells/mL) in PBS with 2 mM EDTA were analyzed using BD LSR Fortessa™ X‐20 (BD) flow cytometer (Becton Dickinson). FITC laser was used to detect CPN positive cells and APC laser detected cells labeled with APC conjugated CD44 antibody. Propidium iodide was used as nuclear control.

### Cell sorting

One million cells were stained with anti CD44 APC-conjugated antibody (BD, #559,942, 20 μl per 10^6^ cells in 100 µl of stain buffer) on ice for 45 min, washed and suspended in PBS/2 mM EDTA at the concentration of 10^6^ cells/mL. The cells were sorted using BD FACSAria Fusion™ cell sorter (BD) to separate CD44 + from CD44- cells and/or Ha-CPN + and HA-CPN- cells.

### Proliferation assay

Cells were seeded at density of 5 × 10^3^ cells per well in 6 well plates. Number of viable cells was assessed at the indicated time points (2-, 4-, and 6-days post-seeding) using trypan blue exclusion assay via hemocytometer counts. Total numbers of viable cells were graphed.

### Neurosphere formation assay

Single cell suspension of GBM patient derived cells was seeded at 5 × 10^4^ in 12 well plates containing 1.5 mL growth media. HA-CPNs were applied at a concentration of 24 CPNs/cell (150µL) and plates were incubated at 37 ºC and 5% CO_2_. After 6 days, spheres were imaged at 10 × magnification under five different fields of view using a Leica inverted microscope (Leica CTR 6500). Primary neurospheres were dissociated in PBS pH 7.4 (ThermoFisher, #10,010–023) and seeded into a secondary generation following the same procedure. ImageJ analysis was used for quantification of sphere diameter and number.

### qRT-PCR

Total RNA was extracted from cells utilizing RNeasyPlus Mini Kit (Qiagen) and reverse transcribed using qScript cDNA SuperMix (Quanta), according to manufacturer instructions. For each experiment, samples were reverse transcribed at the same time and cDNA was stored at − 20 °C. Real time qPCR with SYBR™ Green (Applied Biosystems) fluorescent detection was performed using Viia7 thermocycler (Life Technologies). Data was analyzed using Viia7 software and represented as RQ relative to control.

### Sphere invasion assay

Single cell suspension of 2.5 × 10^4^ human primary cells were seeded in 96 well round bottom ultra-low attachment plates containing 200µL growth media per well. Spheroids were cultured at 37 °C and 5% CO_2_ for 3 days. Individual spheroids were embedded in a 30µL droplet of Matrigel™ (FisherScientific, #CB40234C) mixed prior with HA-CPN volume corresponding to 12–24 HA-CPNs/cell. Embedded spheroids were transferred to 24 well surface repellent plates containing 750µL growth media and imaged every 24 h using a Leica inverted microscope (Leica CTR 6500). The average radius of collective cell migration (4 measurements per sphere, 6 spheres per treatment over 3 patient lines) and single cell migration were measured using ImageJ, starting from the central point of the core in each sphere analyzed. The radius of collective cell migration was corrected by the radius of time 0. ImageJ was used to quantify number of migrating single cells.

### Migration assay

Cells were seeded at 7.5 × 10^4^ into twelve well cell culture inserts with 8 µm porosity polyester membrane filters (VWR, #62,406–176) in 500µL of serum-free EMEM. 1 mL of growth media was added into each well of the plate. After a 24-h incubation period at 37 °C and 5% CO_2_, not migrated cells were removed from the insert using a cotton swab. Migrated cells which crossed the membrane were washed in 1xPBS and fixed in 4% PFA, followed by staining with 0.1% crystal violet in methanol. Rinsed membranes were imaged at 10 × magnification under five different fields of view using a Leica inverted microscope (Leica CTR 6500). ImageJ analysis provided quantification of the signal’s Integrated Density.

### Drug treatment assay

Human primary cells were cultured at 1 × 10^4^ cells per well in 96 well round bottom ultra-low attachment plates containing 100µL growth media. Temozolomide (20 µM) (Selleckchem, #S1237), dinaciclib (15 nM) (Selleckchem, #S2768) and HA-CPNs (24 CPNs/cell) were applied 1-h post-seeding. Respective DMSO and HA-CPN vehicle controls of each drug were used. Plates were incubated at 37 ºC and 5% CO_2_ for 72-h and all treatments were refreshed at half of the originally applied concentration every 24 h throughout incubation period. Cell viability was measured via the CellTiter-Glo 3D Cell Viability Assay (Promega) using Spectramax plate reader.

### MTT assay

Cells were seeded in 96-well plates (5 × 10^3^ per well in 100μL of growth media). The plates were protected from light with aluminum foil and incubated at 37 °C and 5% CO_2_. At the indicated time points, the media was removed and replaced with 20μL of MTT solution (5 mg/mL thiazolyl blue tetrazolium bromide along with filter-sterilized PBS) (Sigma Aldrich, #M5655) and the plates were incubated for 2–4 h at 37 °C and 5% CO_2_. This was followed by the addition of 100μL of extraction buffer. The plates were incubated overnight at 37 °C and 5% CO_2_ and protected from light. The absorbance at 590 nm was measured using SpectraMax plate reader.

### Dinaciclib-mediated CD44 enrichment and CPN uptake analysis

Cells (2.5 × 10^4^) were seeded in 6 cm plates and treated with 15 nM dinaciclib (Selleckchem, #S2768) or DMSO (as a vehicle control), and 150 µL of CPNs for 72-h at 37 °C and 5% CO_2_ while protected from light. The media was replaced on each of these plates, along with the addition of drugs and CPNs every 24 h. At 72 h the cells were subjected to flowcytometry analysis.

### Immunocytochemistry analysis

The cells were washed twice with 1xPBS, followed by fixing in 4% PFA and staining with anti- CD44 antibody (Novus, #NBP1-31,488) when required, for 2 h. The cells were then washed with 1xPBS and HBSS and incubated with fluorophore conjugated secondary antibody (ThermoFisher, #A-11011) for 1 h and Hoechst nuclear stain for 15 min. The coverslips were washed for 5 min each, with HBSS, 1xPBS, and diH2O. The coverslips were mounted on microscope slides using aqueous mounting media and imaged using a Leica inverted microscope (Leica CTR 6500 microscope).

### Endocytosis inhibitor treatment and protein expression analysis

Endosidin 9 (#SML2726, Sigma) was added to cell culture media in final concentration of 30 μm for 1–2 h prior the addition of HA-CPNs. The cells were incubated for 24 h and collected for lysis and protein expression analysis using SDS-PAGE. Antibodies, anti CD44 (# NBP1-47,386, Novus) and anti- CD44-ICD (#KAL-KO601, CosmoBio) were used at concentrations of 1:1000 and 12 μm/mL, respectively.

### Zebrafish injections and PDX models

Zebrafish embryos at 3dpf were anesthetized and positioned on the 1% agarose. Cells (10^6^ cells/mL) were labeled with Vibrant™ DiI and DiD cell tracing dyes (ThermoFisher, # V22885, V22887) and 9.2 nl of cell suspension in the presence of CPNs (4 CPNs/cell) or vehicle control were injected into the midsection of the yolk sac using Nanoject II Auto- Nanoliter Injector (Drummond). The embryos were imaged at the indicated timepoints using Leica fluorescence stereomicroscope M205. Analysis of tumour foci burden was measured, and Integrated Density of the signal was quantified using ImageJ software. The values obtained from CPN treatment were corrected by the vehicle control values at each time point.

## Supplementary Information

Supplementary Information.

## Data Availability

The datasets used and/or analysed during the current study available from the corresponding author on reasonable request.
