# Supplementary material for: Impairing proliferation of glioblastoma multiforme with CD44+ selective conjugated polymer nanoparticles
Source: Sci Rep. 2022 Jul 15;12:12078. doi: 10.1038/s41598-022-15244-0 (PMC9287456; doi:10.1038/s41598-022-15244-0)
Supplement: Supplementary file 1 — Supplementary Information. [file 41598_2022_15244_MOESM1_ESM.docx]

**Supplementary Information**

**Impairing Proliferation of Glioblastoma Multiforme with CD44+ Selective Conjugated Polymer Nanoparticles**

Dorota Lubanska, ^1, ‡^ Sami Alrashed,^1, ‡^ Gage T. Mason,^2, ‡^ Fatima Nadeem,^1^ Angela Awada,^2^ Mitchell DiPasquale,^2^ Alexandra Sorge,^1^ Aleena Malik,^2^ Monika Kojic,^2^ Mohamed A.R. Soliman,^3, 4, 5^ Ana C. deCarvalho,^6^ Abdalla Shamisa,^5^ Swati Kulkarni,^5^ Drew Marquardt,^2,7^ Lisa A. Porter ^1,^ * and Simon Rondeau-Gagné ^2,^ *

^1^ Department of Biomedical Sciences, University of Windsor, 401 Sunset Ave. Windsor, Ontario, Canada N9B 3P4 ^2^ Department of Chemistry and Biochemistry, University of Windsor, 401 Sunset Ave. Windsor, Ontario, Canada N9B 3P4

^3^ Department of Neurosurgery Faculty of Medicine Cairo University Cairo, Egypt

^4^ Department of Neurosurgery, Jacobs School of Medicine and Biomedical Sciences, University at Buffalo, Buffalo, NY, U.S.

^5^ Schulich School of Medicine and Dentistry, Western University, London, ON, Canada

^6^ Department of Neurosurgery, Henry Ford Hospital, Detroit, MI 48202, USA

^7^ Department of Physics, University of Windsor, 401 Sunset Ave. Windsor, Ontario, Canada N9B 3P4

^‡^ These authors have equally contributed to this work

* Co-corresponding authors

**Reagents for chemical synthesis.** Chemical reagents were purchased from Sigma-Aldrich Co. Canada, Matrix Scientific, or Oakwood Products Inc. and were used as received. Solvents used for organic synthesis were obtained from Sigma-Aldrich and purified with a Solvent Purifier System (SPS) (Swagelok, Solon, Ohio, USA). Tris(dibenzylideneacetone)dipalladium(0)-chloroform adduct (Pd_2_(dba)_3_•CHCl_3_) was purchased from Sigma Aldrich and recrystallized following a reported procedure ^1^. Fluorescein-labelled Hyaluronic Acid (*M*_w_ = 6 kDa, degree of substitution = 0.001-0.008 ) was purchased from TdB Labs and used without further purification.

**Preparation of the control Nc-CPNs.** The control nanoparticles, without hyaluronic acid were prepared *via* nanoprecipation. 1 mg of P(DPP-T) conjugated polymer and 2 mg of Tween80 were dissolved in 1 mL of tetrahydofuran (THF) and left to stir for 30 minutes. The solution was then injected in 6 mL of deionized water under probe sonication (amplitude of 60, power of 55W for 5 minutes). After sonication, the sample was passed through a 0.2µm PTFE (hydrophobic) syringe filter to remove any large aggregated materials. The nanoparticles were washed multiple times with deionized water and the resulting aqueous suspension was stored at 4°C prior to use. Analysis by dynamic light scattering (DLS) confirmed the formation of nanoparticles, with an average diameter of 81 nm and a dispersity of 0.487.

**Measurements and characterization of HA-CPNs.** Dynamic Light Scattering measurements were obtained using a Zetasizer Nano ZS utilizing a He-Ne laser at wavelength 633nm and a power of 100VAC at 25°C. A multi-laser nanoparticle tracking analysis system (Horiba ViewSizer 3000) was used to determine the nanoparticle concentration at 1 mL. Small angle neutron scattering (SANS) measurements were conducted on the NGB 30 m SANS instrument located at the National Institute of Standards and Technology Center for Neutron Research (NCNR, Gaitherburg, MD) ^2^. Samples were loaded into 1 mm path-length quartz banjo cells and mounted in a cell holder thermally-regulated by a circulating water bath with ≈1 °C accuracy. Measurements were recorded at 35 ºC using a neutron wavelength of 6 Å, as well as three sample-to-detector distances (1.33 m, 4 m, and 13.2 m), to access a scattering vector range of approximately 0.003 Å^−1^ to 0.5 Å^−1^. The scattered beam was counted on a 2D ^3^He detector and radially- averaged to produce 1D scattering curves of total intensity against the scattering vector (I vs. Q). Data were reduced and stitched using Igor Pro and NCNR developed reduction scripts to account for detector sensitivity, sample transmission, and background scattering from heavy water ^3^. SANS data were analyzed using the openly available SASView (<http://www.sasview.org/>) by fitting the shape-independent Guinier-Porod model to define the size and dimensionality of the scattering particle, as described by Hammouda ^4^. Fit parameters are provided in Table S1, and particle radius is extracted as $R= {R_{g}}/{\left( \frac{3}{5} \right)^{0.5}}$. Nuclear magnetic resonance (NMR) spectra were recorded on Bruker NMR spectrometers (DPX 300 and DPX 300 US operating at 300 MHz (^1^H) and 75 MHz (^13^C)). UV-vis spectroscopy was performed on a Varian UV/Visible Cary 50 spectrophotometer. Thermal gravimetric analysis was performed on a Mettler Toledo TGA SDTA 851e. Nitrogen (99.999%) was used to purge the systems at a flow rate of 60 mL/min. All samples were run in aluminum crucibles. TGA samples were held at 25 °C for 30 min before heated to 550 °C at a rate of 5 °C/min. FTIR spectroscopy was performed on a Bruker ALPHA FTIR Spectrometer using a Platinum ATR sampling module. Electron microscopy was performed at the Canadian Centre for Electron Microscopy (also supported by NSERC and other government agencies).

| **Table S1.** Fit parameters of Guinier-Porod model for HA-CPNs from SASView. | |
| --- | --- |
| Scattering Vector Continuity *q_con_* | 0.0077 Å^-1^ |
| Radius of Gyration *R_g_* | 32.6 ± 0.8 nm |
| Porod exponent *d* | 4.2 ± 0.02 |
| Dimensionality *s* | 0.02 ± 0.06 |
| Shape Radius *R* | 42.1 ± 1.1 nm |

**Synthesis of P(DPP-T)**

**Figure S1.** Synthetic pathway for **P(DPP-T)**.

**Synthetic procedure for the preparation of P(DPP-T).** A microwave vessel equipped with a stir bar was charged with 3,6-bis(5-bromothiophen-2-yl)-2,5-bis(2-decyltetradecyl)-2,5-dihydropyrrolo[3,4-c]pyrrole-1,4-dione (63.7 mg, 0.052 mmol), 2,5-bis(trimethylstannyl)thiophene (25.0 mg, 0.061 mmol), degassed chlorobenzene (2.1 mL), Pd_2_(dba)_3_ (1.10 mg, 0.001 mmol) and P(*o*-tolyl)_3_ (1.70 mg, 0.005 mmol) and degassed with N_2_ for 30 minutes. The vessel was then immediately sealed with a snap cap and microwave irradiated under the following conditions with ramping temperature (Microwave Setup: Biotage Microwave Reactor; Power, 300 W; Temperature and Time, 2 minutes at 100^o^C, 2 minutes at 120 ^o^C, 5 minutes at 140 ^o^C, 5 minutes at 160 ^o^C, and 40 minutes at 180 ^o^C; Pressure, 17 bar; Stirring, 720). After completion, the polymer was end-capped with **trimethylphenylstannane** (14.7 mg, 0.061 mmol) and bromobenzene (9.60 mg, 0.061 mmol), successively. The reaction was then cooled to room temperature and dissolved in 1,1,2,2-tetrachloroethane. This solution was then precipitated in methanol and the solid was collected by filtration into a glass thimble. The content of the thimble was then extracted in a Soxhlet extractor with methanol, acetone, hexane. The hexane fraction was concentrated and reprecipitated in methanol, followed by filtration and drying under vacuum (yield = 73%). Molecular weight estimated from high-temperature GPC: *M*_n_ = 13.6 kDa, *M*_w_ = 21.1 kDa, PDI = 1.55


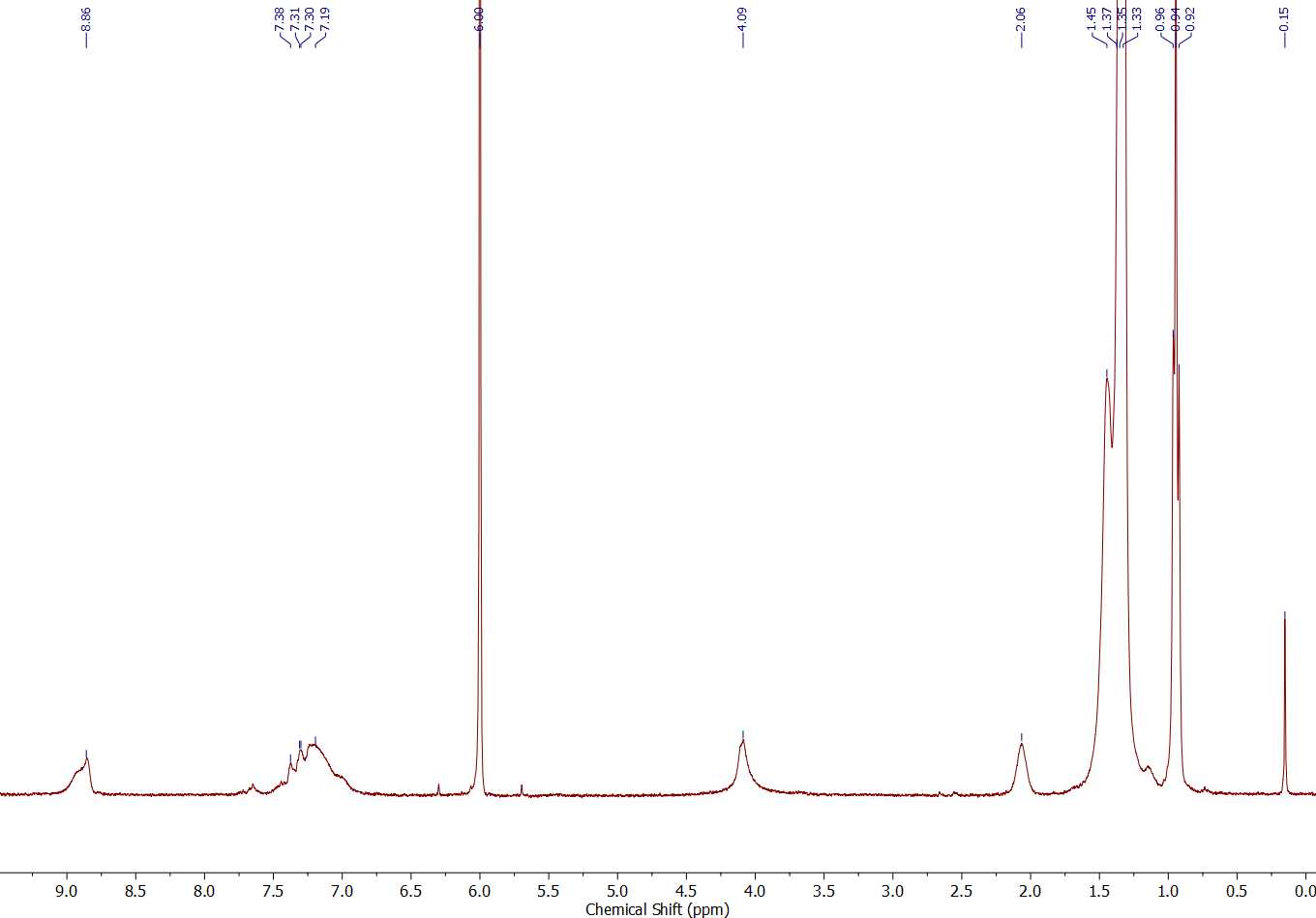


**Figure S2.** ^1^H NMR spectrum of **P(DPP-T)** in 1,1,2,2-tetrachloroethane-*d*_2_ at 100°C.


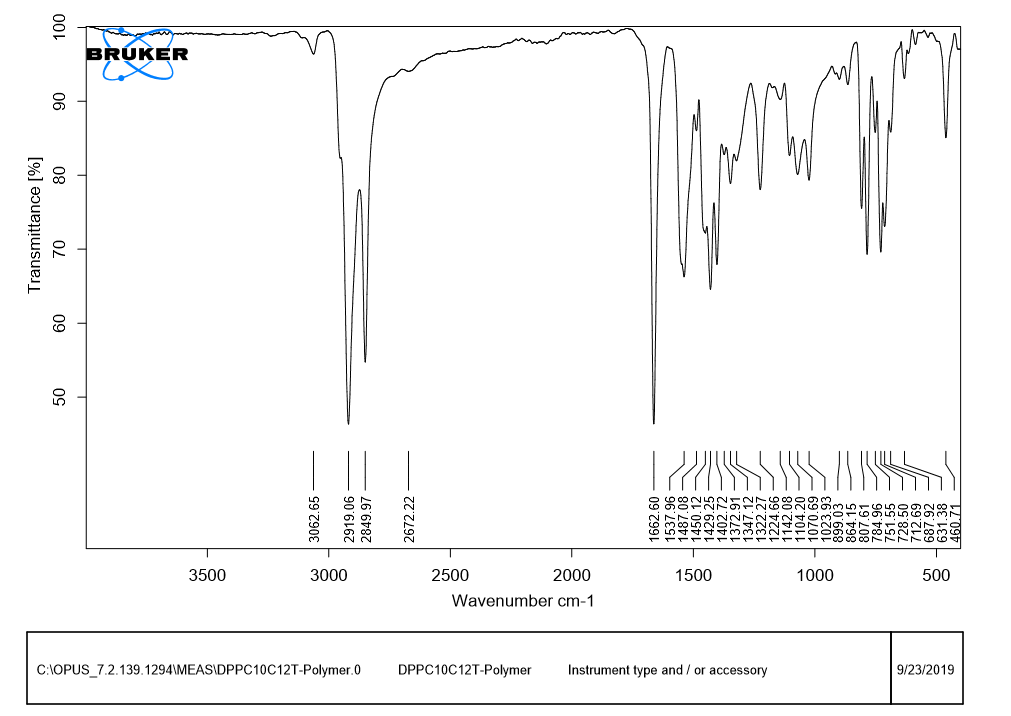


**Figure S3.** Fourier-Transform Infrared spectra (FTIR) of P(DPP-T) in thin film.

**Table S1.** Stability of HA-CPNs in water, 10% fetal bovine serum (FBS) and phosphate buffer (PBS) as verified by Zeta potential analysis.

|  | **Day 1** | **Day 5** |
| --- | --- | --- |
| **HA-CPN** | -37.0 | -38.1 |
| **HA-CPN in 10% FBS_(aq)_** | -26.3 | -31.3 |
| **HA-CPN in PBS_(aq)_** | -40.7 | -38.5 |


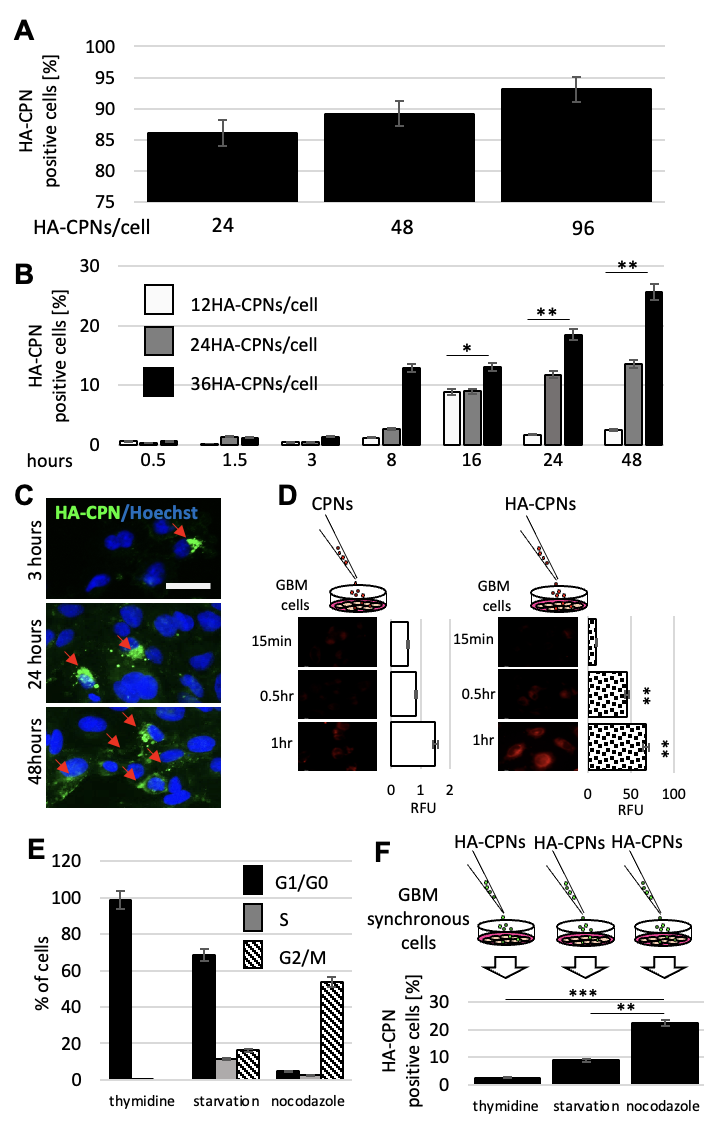


**Figure S4. Optimization of concentration and timing of HA- CPN treatment *in vitro*.** **a** Flowcytometry of HA-CPN uptake at 96 hours by U-251 MG cells treated with higher concentrations of nanoparticles – 24, 48, 96 HA-CPNs/cell; **b** Uptake of HA-CPNs over 48 hours assessed by flowcytometry at the indicated time points for concentrations of 12, 24, and 36 particles/cell. **c** Subcellular localization of HA-CPNs incorporated by U-251 MG cells monitored at the indicated time points. Scale bar = 40μm; **d** Quantified fluorescence (RFU) in U251 MG cells treated with HA-CPNs and CPNs without conjugated HA, at the indicated time points using a plate reader; **e** Different cell cycle synchronization conditions and number of cells in each phase quantified as % of total cell number; **f** HA-CPN uptake in relation to the synchronized populations, quantified as % of total cell number. Data shown as mean ± s.d, n=3, ***p<0.001; Student’s *t*-test. Data shown as mean ± s.d, n=3, *p<0.05, **p<0.01, ***p<0.001; Student’s *t*-test.


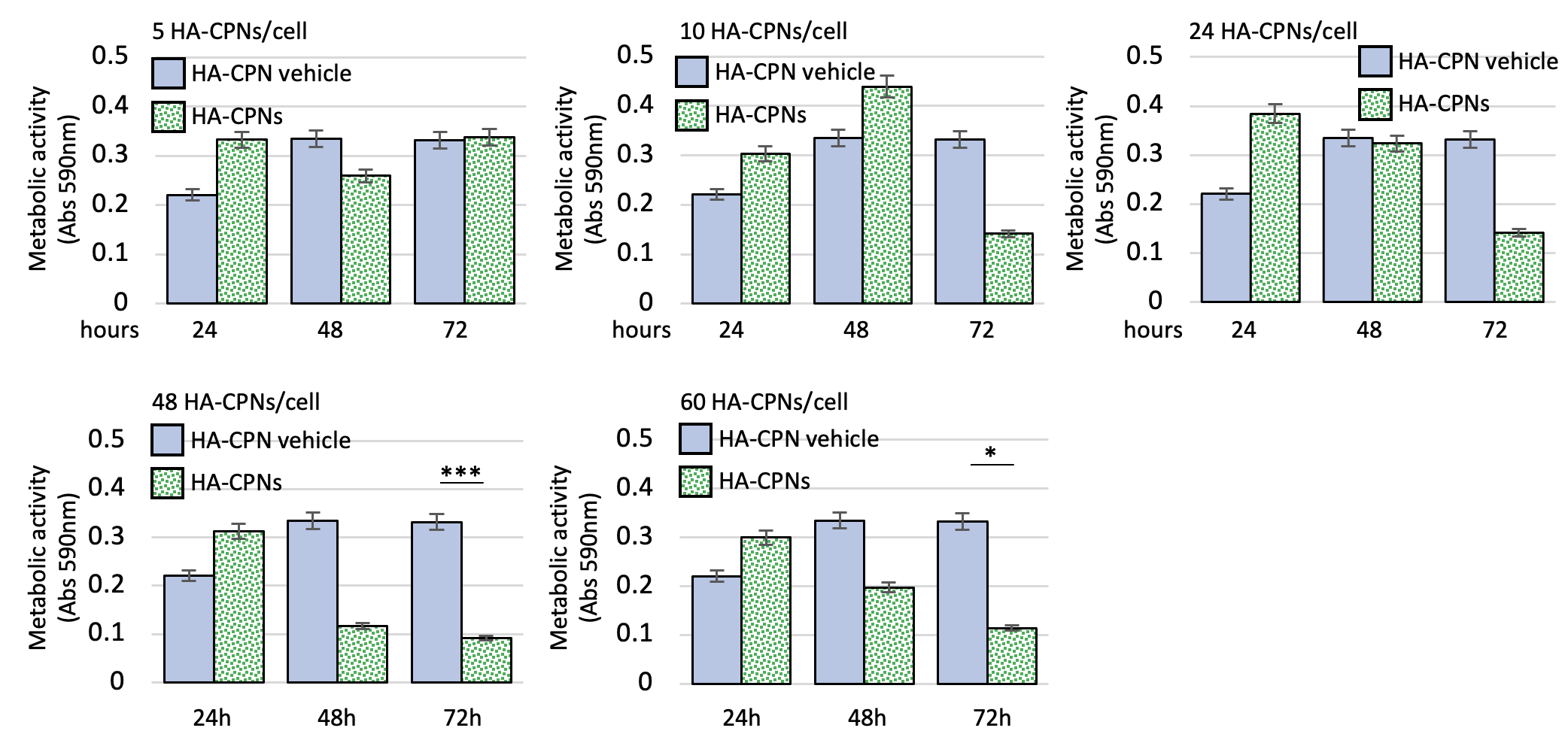


**Figure S5. HA- CPN Treatment Affects Metabolic Activity of Glioma.** U-251 MG cells treated with indicated concentrations of HA-CPNs or vehicle control (Control) and subjected to a 72- hour MTT assay time course. Data shown as mean ±s.d, n=3, *p<0.05, ***p<0.001; Student’s *t*-test.

**
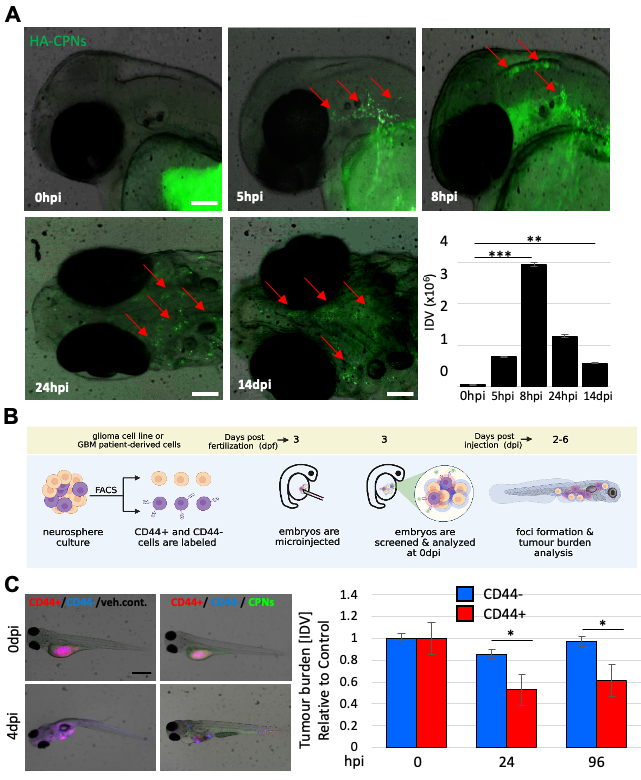
**

**Fig. S6 Blood Brain Barrier Permeability and Effects of HA-CPNs on GBM *in vivo.*** **a** HA-CPN blood-brain barrier penetration in zebrafish over a time course of 14 days. Zebrafish injected with 9.2nl of HA-CPNs at 0dpi and imaged at the indicated time points. HA-CPNs detected in the head/brain regions (left) and specific fluorescence quantified as IDV (Integrated Density Values) using ImageJ (right). Scale bar =100μm; **b** Schema of the workflow and timing of zebrafish GBM PDX models (built from Biorender); **c** Representative images (left) and tumour foci burden analysis (right) in zebrafish embryos co-injected with fluorescently labelled CD44+ (red) and CD44- (blue) cells and analysed at 0, 24 and 96 hours post injection. Tumour burden quantified and graphed as Integrated Density Values (IDV) for HA- CPN treatment/ HA-CPN vehicle control treatment (Control) at the indicated time points. Scale bars: 500μm. Data shown as mean ± s.d, n=3, *p<0.05, **p<0.01; Student’s *t*-test.


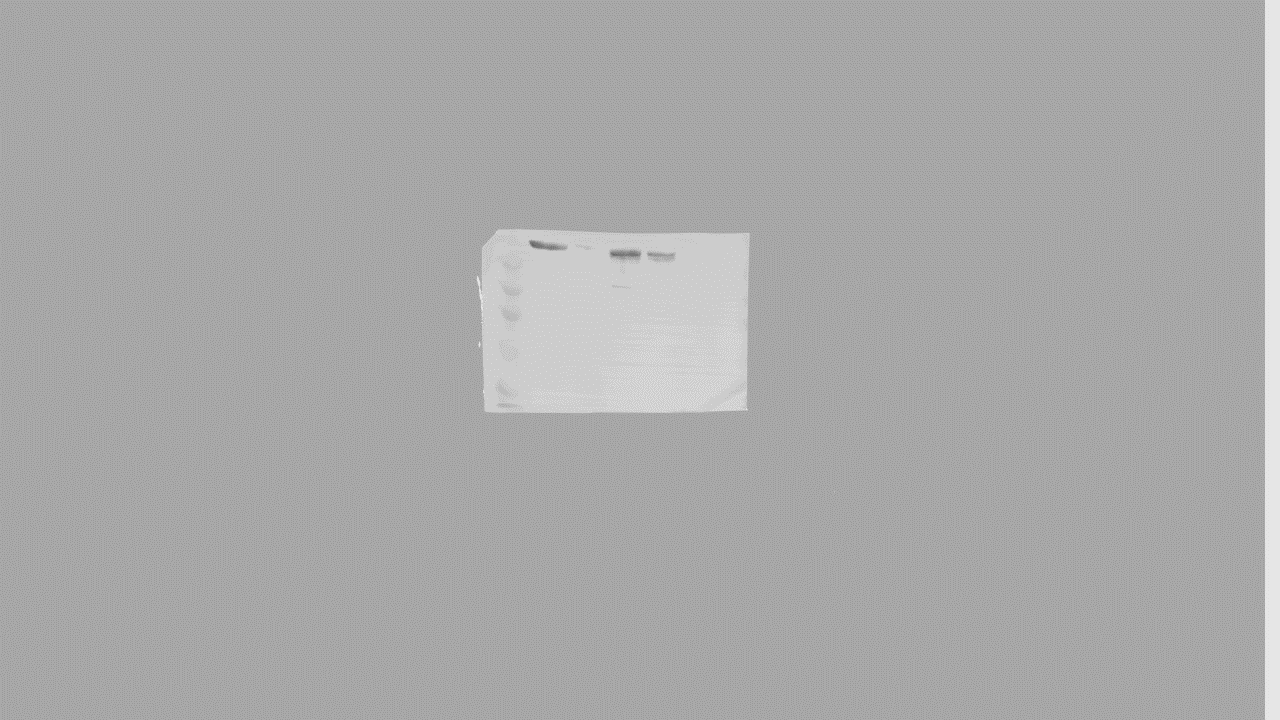

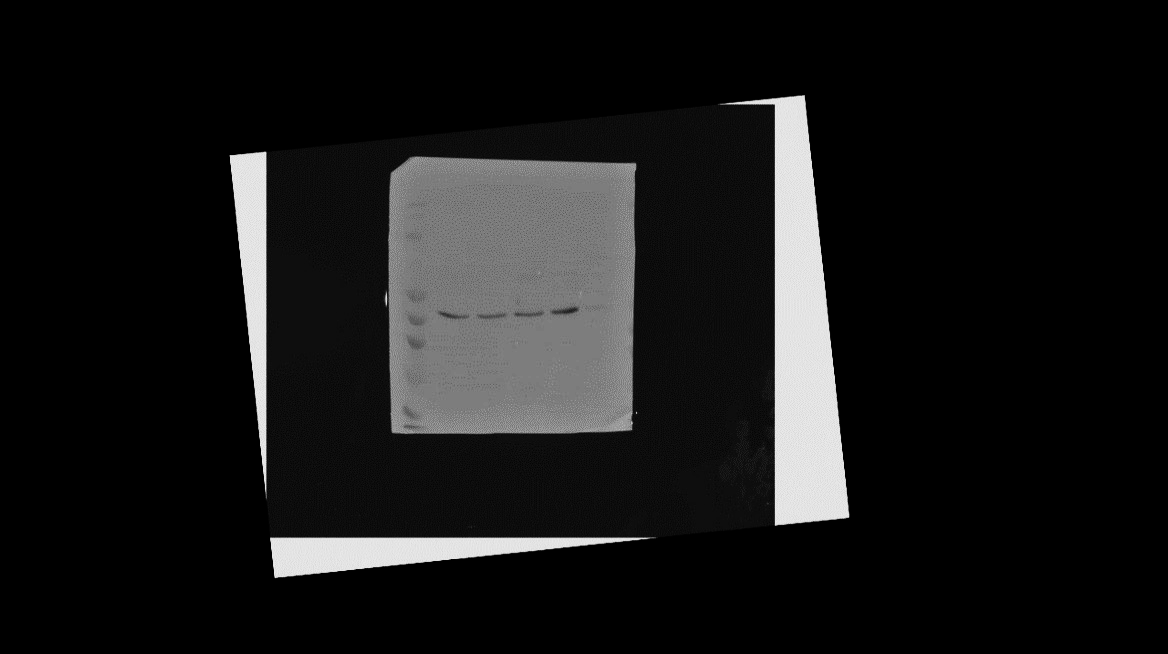

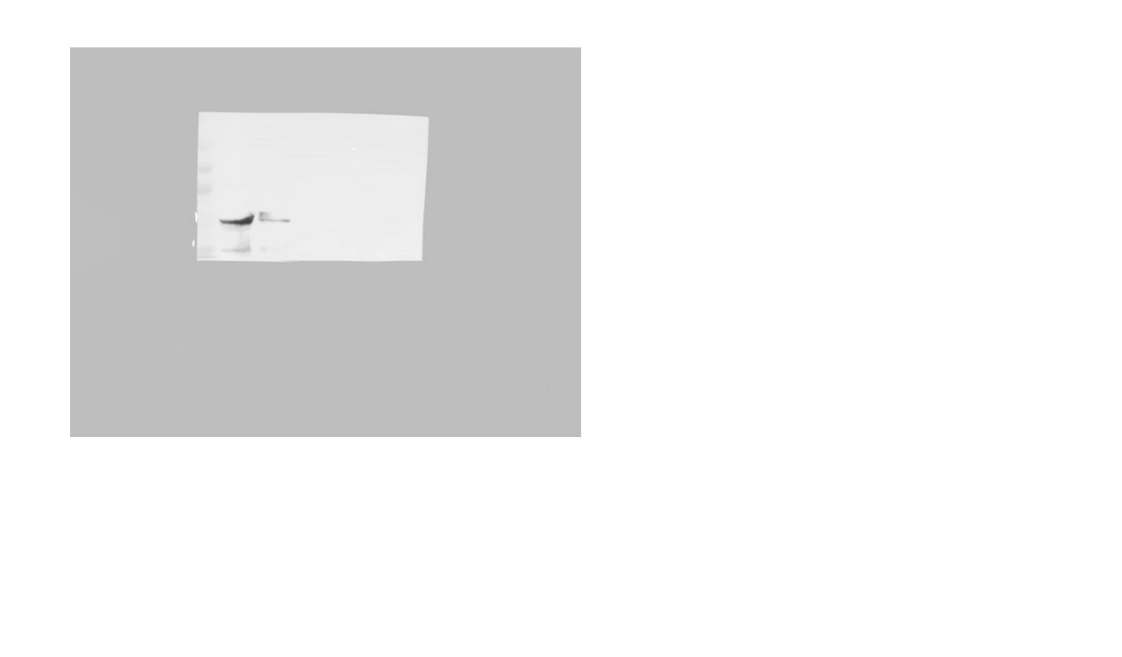

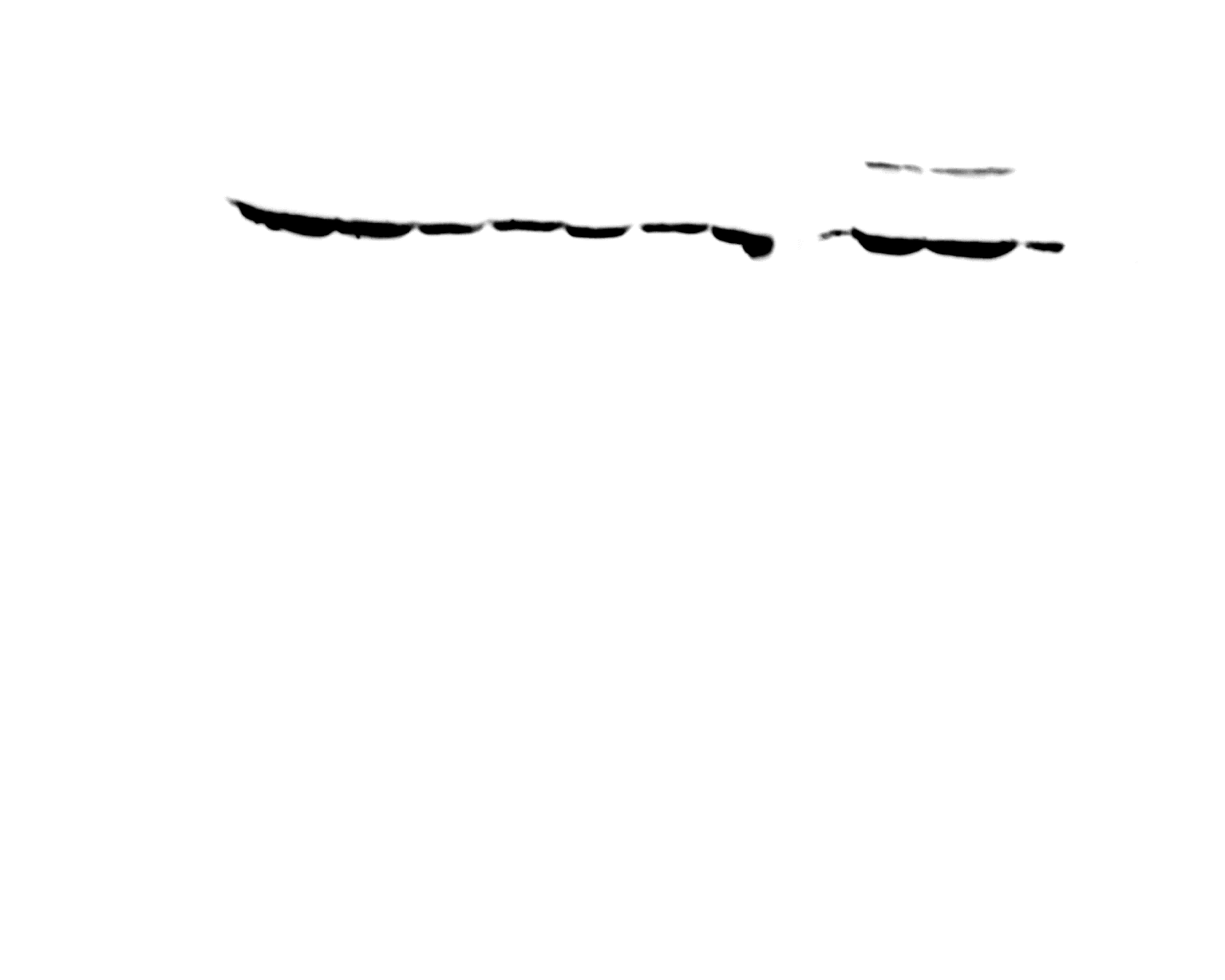

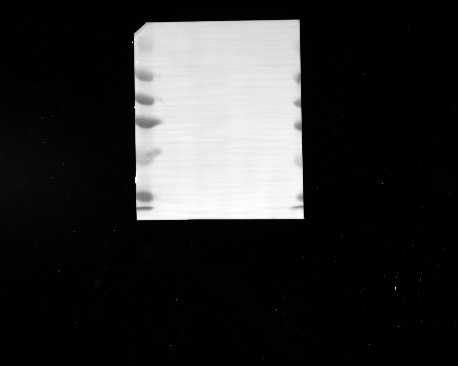

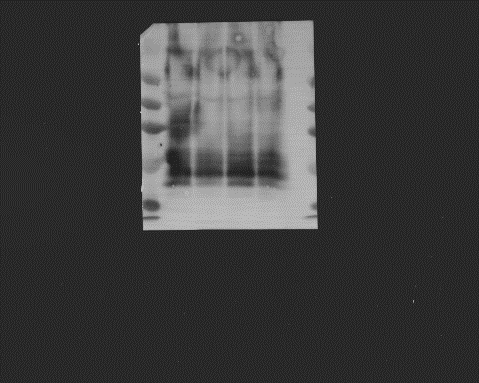

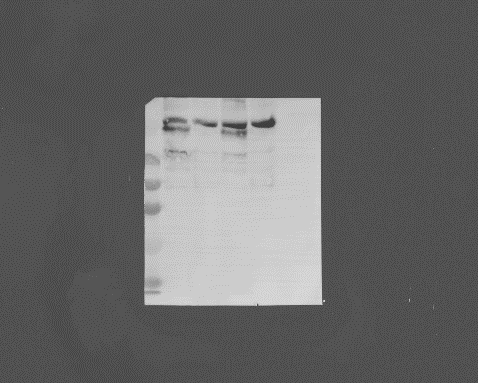


Figure 5B

**CD44**

**82kDa**

**Actin**

Figure 5D

**CD44**

**82kDa**

**CD44**

**-ICD**

**16.5kDa**

**CD44**

**-ICD**

**16.5kDa**

**Actin**

**Fig. S7** Original blotted membranes of Western blot for Figure 5B and 5D. The molecular weight of proteins of interest, CD44 and CD44-ICD, are indicated. Boxed are bands of the blot presented in the Figure 5B.

**References**

1. Zalesskiy, S. S. & Ananikov, V. P. Pd_2_(dba)_3_ as a precursor of soluble metal complexes and nanoparticles: Determination of palladium active species for catalysis and synthesis. *Organometallics* **31**, 2302–2309 (2012).

2. Glinka, C. J. *et al.* The 30 m Small-Angle Neutron Scattering Instruments at the National Institute of Standards and Technology. *J. Appl. Crystallogr.* **31**, 430–445 (1998).

3. Kline, S. R. Reduction and analysis of SANS and USANS data using IGOR Pro. *J. Appl. Crystallogr.* **39**, 895–900 (2006).

4. Hammouda, B. A new Guinier-Porod model. *J. Appl. Crystallogr.* **43**, 716–719 (2010).
